# Management of colorectal cancer liver metastasis in a patient with immune thrombocytopaenia

**DOI:** 10.1308/003588413X13511609957498

**Published:** 2013-03

**Authors:** M Yacob, RS Raju, FL Vyas, P Joseph, V Sitaram

**Affiliations:** Christian Medical College, Vellore,India

**Keywords:** Immune thrombocytopaenia, Hepatectomy, Carcinoma rectum

## Abstract

Immune thrombocytopaenia (ITP) was referred to previously as idiopathic thrombocytopaenic purpura and is usually of autoimmune or viral aetiology. Colorectal cancer liver metastasis with concomitant ITP is rare and only three cases have been reported in the English literature. Adverse effects of adjuvant chemotherapy may aggravate ITP. The sequencing of chemotherapy, operation for the primary and liver metastasis, and a decision on splenectomy is important. We present our experience in the management of a 52-year-old man who, having undergone anterior resection one year earlier for carcinoma of the rectum, presented with liver metastasis and ITP. He underwent splenectomy with hepatectomy prior to chemotherapy.

Immune thrombocytopaenia (ITP) has many causes. Common causes include viral infection and autoimmune disease.^1^ Colorectal cancer with liver metastasis and ITP is a rare clinical scenario; only three cases have been reported in the English literature.^2,3^ Adverse effects of adjuvant chemotherapy for liver metastasis may aggravate ITP.^4^ The sequencing of chemotherapy, operation for the primary and liver metastasis, and splenectomy is important.

## Case history

A 52-year-old man had undergone low anterior resection for a T3N1M0 carcinoma of the rectum at another centre a year previously. Adjuvant chemotherapy was deferred owing to low platelet counts. He was referred to us for further management. Clinical examination was unremarkable except for the scar of the previous operation.

**Figure 1:**
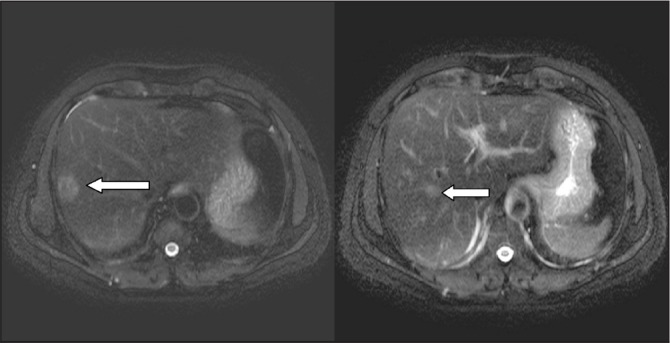
Magnetic resonance imaging showing liver metastasis in segments V and VII/VIII of the right lobe of the liver

Blood investigations were normal except for a platelet count of 63,000/mm^3^. Magnetic resonance imaging of the abdomen revealed hepatomegaly with a 2.5cm × 2.5cm lesion in the subcapsular region of segment VIII on the lateral aspect and a 1.5cm × 1cm lesion in segment V (Fig 1). The patient was evaluated by haematology and was diagnosed with ITP. Chemotherapy was deferred owing to the low platelet count. He was scheduled for a right hepatectomy and splenectomy.

Intraoperatively, there was one lesion each in segments VIII and V; the second lesion was close to the gallbladder bed. Findings were confirmed with on-table ultrasonography. The patient underwent a splenectomy and right hepatectomy. He made an uneventful recovery and his platelet count improved. He then received six cycles of adjutant chemotherapy with 5-fluoro uracil, oxaliplatin and leucovorin. The platelet count did not come down during chemotherapy. He has remained disease-free and asymptomatic for the last ten months.

## Discussion

ITP is one of the causes of thrombocytopaenia.^1^ It is an acquired immune mediated disorder characterised by isolated thrombocytopaenia in the absence of other causes of thrombocytopaenia.^5^ An international working group divided ITP into primary ITP, where other conditions associated with thrombocytopaenia are absent, and secondary ITP, which can be due to bacterial or viral infection, and underlying autoimmune and lymphoproliferative disorders.^1^

The diagnosis of ITP is made by excluding other causes of thrombocytopaenia. Low platelet count and the presence of antiplatelet antibodies confirm the diagnosis. Bone marrow examination shows peripheral platelet destruction, and also excludes leukaemia, infiltrative disease and aplastic anaemia. Treatment of ITP includes corticosteroids and intravenous immune globulin for patients unresponsive to corticosteroids. A splenectomy is considered in patients with failed corticosteroid therapy and is considered effective.^3^

ITP in association with carcinoma of the rectum and other gastrointestinal tract malignancies is rare. A few cases of thrombocytopaenia with solid tumours have been reported in the literature.^2^ Treatment of carcinoma of the rectum includes surgical excision and chemoradiotherapy depending on the stage of the disease and grade of tumour. Our patient had undergone anterior resection in another centre. Chemotherapy was deferred owing to the low platelet count. The timing of chemotherapy, liver resection and splenectomy is very important.^4^ In ITP patients with colorectal cancer and liver metastasis, liver resection with a splenectomy followed by chemotherapy is probably the best sequence.

## Conclusions

Colorectal cancer with liver metastasis in patients with ITP is rare. Liver resection and a splenectomy can be performed in patients with resectable colorectal liver metastasis prior to adjuvant chemotherapy.
